# Serum glial fibrillary acidic protein is sensitive to acute but not chronic tissue damage in cerebral small vessel disease

**DOI:** 10.1007/s00415-022-11358-7

**Published:** 2022-09-03

**Authors:** Thomas Gattringer, Christian Enzinger, Daniela Pinter, Simon Fandler-Höfler, Markus Kneihsl, Melanie Haidegger, Sebastian Eppinger, Rina Demjaha, Arabella Buchmann, Andrea Jerkovic, Reinhold Schmidt, Michael Khalil

**Affiliations:** 1 Department of Neurology, Medical University of Graz, Auenbruggerplatz 22, 8036 Graz, Austria; 2 Division of Neuroradiology, Vascular and Interventional Radiology, Department of Radiology, Medical University of Graz, Graz, Austria; 3 Institute of Molecular Biosciences, University of Graz, Graz, Austria

**Keywords:** Glial fibrillary acidic protein, GFAP, Stroke, Lacunar stroke, Cerebral small vessel disease, Recent small subcortical infarcts

## Abstract

**Background:**

Serum glial fibrillary acidic protein (sGFAP) has been proposed as a biomarker in various neurological diseases but has not yet been systematically investigated in patients with cerebral small vessel disease (CSVD). We explored whether sGFAP levels are increased in stroke patients with MRI-confirmed recent small subcortical infarcts (RSSI) and analyzed the subsequent course and determinants of sGFAP longitudinally.

**Methods:**

In a prospectively-collected cohort of stroke patients with a single RSSI (*n* = 101, mean age: 61 years, 73% men), we analyzed brain MRI and sGFAP using a SIMOA assay at baseline and at 3- and 15-months post-stroke. Community-dwelling age- and sex-matched individuals (*n* = 51) served as controls.

**Results:**

RSSI patients had higher baseline sGFAP levels compared to controls (median: 187.4 vs. 118.3 pg/ml, *p* < 0.001), with no influence of the time from stroke symptom onset to baseline blood sampling (median 5 days, range 1–13). At the 3- and 15-months follow-up, sGFAP returned to control levels. While baseline sGFAP correlated with larger infarct size (*r*_*s*_ = 0.28, *p* = 0.01), neither baseline nor follow-up sGFAP levels were associated with chronic CSVD-related lesions (white matter hyperintensities, lacunes, microbleeds) after adjusting for age, sex and hypertension. Furthermore, sGFAP levels did not relate to the occurrence of new vascular brain lesions on follow-up MRI.

**Conclusions:**

sGFAP is increased in patients with CSVD-related stroke and correlates with the size of the RSSI. However, sGFAP levels were not related to chronic neuroimaging features or progression of CSVD, suggesting that sGFAP is sensitive to acute but not chronic cerebrovascular tissue changes in this condition.

## Introduction

Glial fibrillary acidic protein (GFAP), the signature intermediate filament of astrocytes, has been proposed as a promising biomarker in various neurological conditions including traumatic brain and spinal cord injury, multiple sclerosis, neuromyelitis optica spectrum disorders, neurodegenerative diseases (e.g. Alzheimer’s disease) and brain tumors [1, 2]. Recent advances in the development and implementation of highly sensitive assays such as the SIMOA technique have enabled robust quantification of GFAP in the blood and its investigation also in healthy individuals, patients with milder neurological deficits and those in whom lumbar puncture is usually not performed.

Prior studies have also analyzed serum GFAP (sGFAP) in patients with acute stroke and consistently indicated that sGFAP, measured in the hyperacute phase, is substantially higher in intracerebral hemorrhage compared to ischemic stroke [3–5]. In addition, higher sGFAP levels on day one after the onset of ischemic stroke have been associated with worse functional neurological outcome at one year even after adjusting for age, initial clinical stroke severity and infarct size [6].

However, data on sGFAP in cerebral small vessel disease (CSVD) and its clinical acute manifestation lacunar stroke, which is responsible for a quarter of all ischemic strokes, is lacking. To date, there is only one published study that has investigated sGFAP in patients with cerebral autosomal dominant arteriopathy with subcortical infarcts and leukoencephalopathy (CADASIL) [7]. In this work, sGFAP levels were increased in CADASIL patients compared to controls. Interestingly, these were also associated with cerebral microbleeds on brain MRI and predicted incident intracerebral hemorrhage over 3 years of follow-up. However, from this study, it remains unclear if sGFAP is already increased in the acute phase of CSVD-related stroke, as blood sampling for sGFAP measurement was performed > 400 days after stroke symptom onset. Moreover, data on temporal dynamics of sGFAP levels (i.e., their evolution over time) after an acute neurological event in general and particularly after stroke and CSVD is scare.

Together, this knowledge gap prompted us to explore whether sGFAP levels are increased in patients with MRI-confirmed, CSVD-related recent small subcortical infarcts (RSSI) and whether this blood biomarker associates with chronic CSVD features such as white matter hyperintensities (WMH), lacunes, or cerebral microbleeds. Moreover, we particularly aimed to study the subsequent course and determinants of sGFAP in a longitudinal manner, assessed at two follow-up investigations at 3 and 15 months after stroke.

## Methods

### Study participants

Patients were selected from a prospective observational study on lacunar stroke patients with an MRI-confirmed single RSSI, suggestive for the supply area of a small perforating brain artery and corresponding to the clinical findings. Age > 75 years, preexisting disability (modified Rankin Scale (mRS) > 1) and contraindications for repeated MRI were defined as exclusion criteria. Besides a thorough clinical neurological assessment and cerebrovascular workup (duplex sonography of extra- and intracranial brain-supplying arteries, echocardiography, and 24-h Holter ECG), patients underwent blood sampling and brain MRI at baseline and at 3 and 15 months after their index event. Detailed description of the study cohort has been reported elsewhere [8, 9].

As controls, we included community-dwelling age- and sex-matched participants from the Austrian Stroke Prevention Family Study. Inclusion criteria were no history of stroke or dementia and a normal neurologic examination. Controls also received a thorough clinical assessment that included medical history, laboratory evaluation, cognitive testing, and an extended vascular risk factor assessment [10].

### Brain MRI

At baseline, all study patients underwent brain MRI at 1.5T (Siemens MAGNETOM Espree, Siemens Healthcare, Erlangen, Germany) according to a standard protocol for the workup of patients with suspected cerebrovascular events. This included the following sequences: axial T2-weighted fast spin echo (SE), axial T2-fluid-attenuated inversion recovery (FLAIR), sagittal T1-weighted SE, gradient echo T2*-weighted, axial diffusion-weighted single-shot echo planar imaging, and a 3D time-of-flight angiography (TOF). All axial scans had a slice thickness of 5 mm. At both follow-ups, brain MRI was performed on a single 3T scanner (Siemens Healthcare). The protocol included high-resolution structural 3-D images by means of a T1-weighted magnetization-prepared rapid gradient echo sequence with 1-mm isotropic resolution, a FLAIR sequence, diffusion-weighted imaging, and 3D-TOF. Controls underwent brain MRI at the same 3T scanner as patients with identical MRI protocols. All MRI scans were reviewed by a neuroradiological expert (CE) according to the STandards for ReportIng Vascular changes on nEuroimaging (STRIVE) [11], blinded to clinical data. WMH were rated according to the Fazekas scale [12]. In addition, we also calculated a commonly used total small vessel disease score (including the presence of lacunes, microbleeds, moderate-to-severe perivascular spaces and WMH) [13].

Follow-up MRI scans were specifically analyzed with regard to changes in cerebrovascular lesions (such as new infarcts, lacunes and WMH progression) as previously described [8].

### Serum GFAP assessment

Peripheral blood (8 mL) was taken by venipuncture within 13 days after the index RSSI (median time from stroke symptom onset to blood sampling 5 days, range 1–13 days) and at 3 and 15 months after stroke. In controls, blood sampling was done in a standardized manner on the same day as MRI within a maximum time interval of 5 h. Serum was then immediately stored at − 80 °C according to international consensus guidelines [14]. We measured sGFAP with a Single Molecule Array (Simoa®, GFAP single-plex discovery kit Ref.-Number: 102336) on the SR-X platform according to the manufacturer’s instructions (Quanterix, Billerica, MA, USA). The assays’ lower limit of quantification lies at 0.686 pg/mL and the limit of detection lies at 0.211 pg/mL. All of our measurements were above these limits. Furthermore, we ensured reproducibility by conducting our sGFAP measurements in duplicates (mean intra-assay coefficient of variation = 6.9%) and comparability by including two native samples as plate controls in all runs (mean concentration = 73.3 pg/mL and 709.5 pg/mL, inter-assay coefficients of variation = 15% and 12%, respectively). Measurements with an intra-assay coefficient of variation > 0.20 (*n* = 3) were excluded from our analyses.

### Statistical analysis

Demographic, clinical, MRI data and sGFAP levels were analyzed with IBM SPSS Statistics 27. The level of significance was set at 0.05. The Kolmogorov–Smirnov test assessed normality of data distribution. Groups were compared by the *χ*^2^ test (for nominal data), Mann–Whitney *U* test (for non-normally distributed variables), or unpaired *t* test (for normally distributed continuous variables). Correlation analysis was performed with the Spearman correlation. After logarithmic transformation of the target variable sGFAP, we performed multivariable linear regression to analyze the effect of time from stroke symptom onset on sGFAP adjusting for age, sex and RSSI diameter [1]. Binary logistic regression analysis served to assess the potential influence of sGFAP levels on the presence of chronic CSVD markers (more severe WMH according to Fazekas scores 2 or 3, presence of lacunes and microbleeds) additionally controlling for the covariates age, sex and arterial hypertension [2, 7].

### Research ethics

This prospective observational study was approved by the ethics committee of the Medical University of Graz (24–260 ex 11/12). All participants gave written informed consent.

### Data sharing

Data that support the findings of this study are available from the corresponding author upon reasonable request*.*

## Results

The cohort comprised 101 RSSI patients (mean age: 61.2 ± 10.6 years, 73% men). Of those, 98 (97%) completed the entire 15-months study period, while 3 patients only completed the follow-up investigation at 3-months post-stroke. Baseline characteristics including clinical data, vascular risk factors, laboratory and MRI findings from patients as well as controls are shown in Table 1. Four patients had concomitant atrial fibrillation, and one patient had a proximal high-grade vessel stenosis, but these findings did not appear to be causally related to the RSSI.

**Table 1:** Baseline characteristics of lacunar stroke patients compared to age-, sex- and WMH-matched controls

	Patients (*n* = 101)	Controls (*n* = 53)	*p* value
Age, mean (SD), years	61.0±10.6	60.6±10.2	0.81
Men, *n* (%)	74 (73)	36 (68)	0.49
NIHSS, median (range)	2 (0–9)	NA	
Progressive lacunar stroke, *n* (%)	14 (14)	NA	
Hypertension, *n* (%)	77 (76)	33 (62)	0.07
Dyslipidemia, *n* (%)	80 (79)	39 (74)	0.43
Diabetes mellitus, *n* (%)	16 (16)	5 (9)	0.27
Smoking, *n* (%)	42 (42)	11 (21)	0.01
History of stroke, *n* (%)	4 (4)	0 (0)	0.30
Maximal axial RSSI diameter, median (range), mm	11.5 (2.0–23.5)	NA	
Deep WMH Fazekas score, median (IQR)	1 (2)	1 (1)	0.13
Periventricular WMH Fazekas score, median (IQR)	1 (2)	1 (0)	0.26
Severe WMH Fazekas scores 2–3, *n* (%)	42 (42)	21 (40)	0.81
Lacunes, *n* (%)	36 (36)	5 (9)	< 0.001
Prior cortical infarcts, *n* (%)	8 (8)	2 (4)	0.50
Cerebral microbleeds, *n* (%)	14 (14)	6 (11)	0.66
Enlarged perivascular spaces in basal ganglia, *n* (%)	31 (31)	NA	
Total small vessel disease score (0–4), median (IQR)	1 (2)	NA	
Stroke symptom onset to baseline blood sampling, median (range), days	5 (1–12)	NA	
sGFAP at baseline^a^, median (IQR), pg/ml	187.4 (232.98)	118.3 (82.2)	< 0.001
sGFAP at 3-month poststroke^b^, median (IQR), pg/ml	119.4 (95.88)	NA	0.78*
sGFAP at 15-month poststroke^c^, median (IQR), pg/ml	115.15 (93.68)	NA	0.85*

*SD* standard deviation, *NIHSS* National Institutes of Health Stroke Scale, *RSSI* recent small subcortical infarcts, *WMH* white matter hyperintensities, *IQR* interquartile range, *sGFAP* serum glial fibrillary acidic protein, *NA* not available

^a^Available in *n* = 90

^b^Available in *n* = 90

^c^Available in *n* = 88

*Compared to GFAP levels in controls

### Baseline sGFAP in RSSI patients vs. controls

**Fig. 1:**
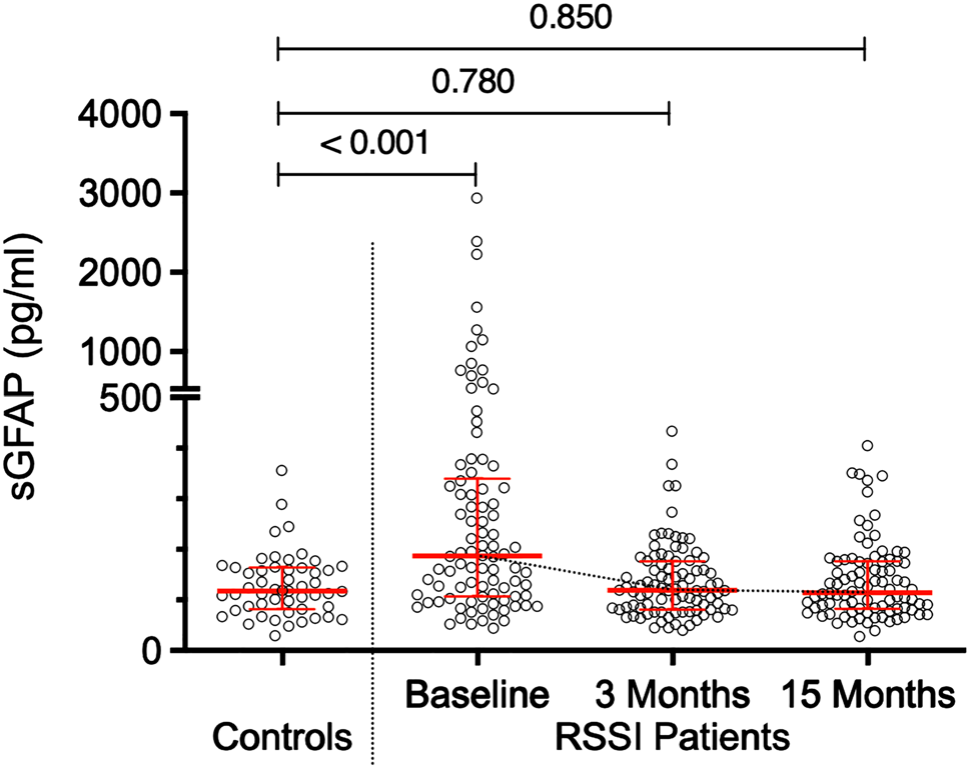
Serum GFAP levels of RSSI patients at baseline, 3- and 15-months follow-up after stroke, and in age- and sex-matched controls

**Fig. 2:**
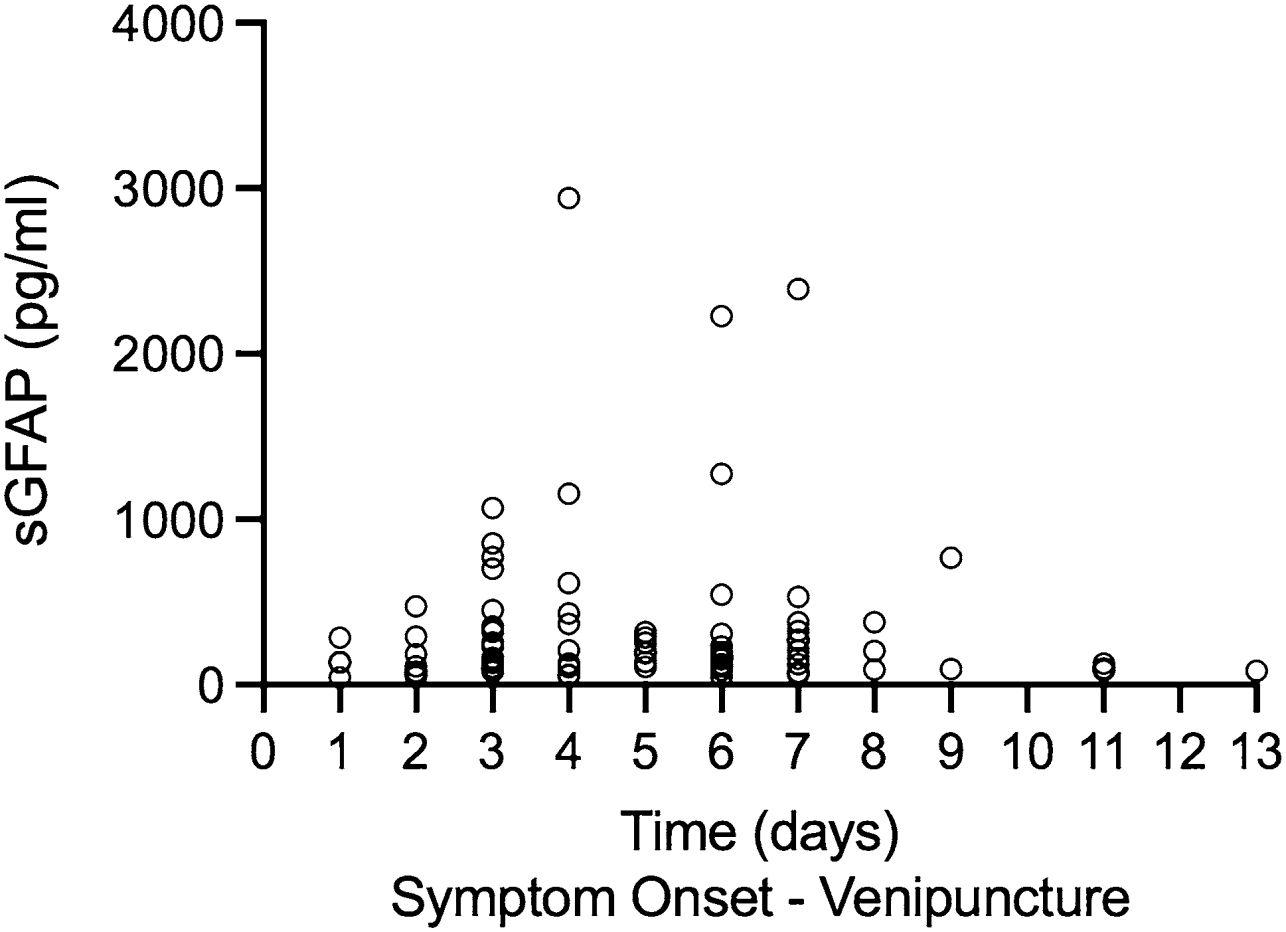
GFAP levels at baseline in relation to time from stroke symptom onset to blood sampling (*r*_*s*_ = − 0.01, *p* = 0.96)

Baseline sGFAP measurements were available for 90/101 patients. Patients with an RSSI had higher baseline sGFAP levels compared to controls (median 187.4 [interquartile range (IQR) 232.9] vs. 118.3 [IQR 82.2] pg/mL, *p* < 0.001, Table 1, Fig. 1). Individual sGFAP levels did not relate to the time from stroke symptom onset to baseline blood sampling within 13 days (median 5 days, range 1–13 days, *r*_*s*_ = − 0.01, *p* = 0.96, Fig. 2). Such an effect was also not observed after adjustment for age, sex and RSSI diameter (*p* = 0.70).

Baseline sGFAP levels were higher in patients with larger RSSI diameter (*r*_*s*_ = 0.28, *p* = 0.01), but did not correlate with clinical stroke severity according to the NIHSS (*r*_*s*_ = 0.173, *p* = 0.10). Neither deep (*r*_*s*_ = 0.07, *p* = 0.51), periventricular (*r*_*s*_ = − 0.05, *p* = 0.67), severe total WMH scores (Fazekas grades 2–3 vs. 0–1: median GFAP: 188.5 vs. 172.1 pg/ml, *p* = 0.62) nor enlarged basal ganglia perivascular spaces (*r*_*s*_ = − 0.02, *p* = 0.85) were associated with baseline sGFAP values. Moreover, sGFAP levels did not significantly differ between patients with presence of cerebral microbleeds (median GFAP: 207.8 vs. 185.8 pg/ml, *p* = 0.65) or lacunes (median sGFAP: 175.5 vs. 187.2 pg/ml, *p* = 0.86), compared to those without these lesions. We also could not identify an association between baseline sGFAP and the total SVD score (*r*_*s*_ = − 0.06, *p* = 0.55).

Patients with prior cortical infarcts had higher sGFAP levels, albeit this was not statistically significant (median GFAP: 368.3 vs. 190.1 pg/ml, *p* = 0.67).

Clinical and MRI characteristics of RSSI patients dichotomized by median levels of baseline sGFAP are displayed in Table 2. When additionally analyzing patients with highest baseline sGFAP levels (quartile 4, GFAP > 340.3 pg/ml, *n* = 22), these had larger RSSI diameters compared to patients with baseline sGFAP levels in quartiles 1–3 (*n* = 68; RSSI diameter: 14.9 vs. 10.3 mm, *p* < 0.001).

**Table 2:** Clinical and MRI characteristics of RSSI patients dichotomized by median levels of baseline serum GFAP

	Baseline sGFAP ≤ 187.4 pg/ml (*n* = 45)	Baseline sGFAP > 187.4 pg/ml (*n* = 45)	*p* value
Age, mean (SD), years	58.9 (10.6)	63.6 (10.6)	0.039
Men, *n* (%)	36 (80)	29 (64.4)	0.1
Hypertension, *n* (%)	34 (75.6)	36 (80)	0.6
Dyslipidemia, *n* (%)	40 (88.9)	32 (71.1)	0.035
Diabetes mellitus, *n* (%)	6 (13.3)	10 (22.2)	0.27
Smoking, *n* (%)	26 (57.8)	11 (24.4)	0.001
History of stroke, *n* (%)	1 (2.2)	3 (6.7)	0.6
NIHSS, median (IQR)	2 (2)	3 (4)	0.32
Progressive lacunar stroke, *n* (%)	5 (11)	9 (20)	0.25
Maximal axial diameter of RSSI (median, IQR, mm)	11.5 (6)	11.5 (6)	0.32
Deep WMH Fazekas score, median (IQR)	1 (1)	1 (3)	0.4
Periventricular WMH Fazekas score, median (IQR)	1 (2)	1 (3)	0.74
Severe WMH Fazekas scores 2–3, *n* (%)	18 (40)	21 (46.7)	0.52
Lacunes, *n* (%)	15 (33.3)	16 (35.6)	0.82
Prior cortical infarcts, *n* (%)	3 (6.7)	5 (11.1)	0.71
Cerebral microbleeds, *n* (%)	6 (13.3)	7 (15.6)	0.76
Enlarged perivascular spaces in basal ganglia, *n* (%)	1 (1)	1 (1)	0.55
Total small vessel disease score (0–4), median (IQR)	1 (2)	1 (2)	0.52
Progressive cerebrovascular disease^a^, *n* (%)	9 (20)	5 (11.1)	0.38
mRS at hospital discharge, median (IQR)	1 (0)	1 (1)	0.1
mRS at 3 months, median (IQR)	1 (1)	1 (1)	0.91
mRS at 15 months, median (IQR)	1 (1)	1 (1)	0.74

*SD* standard deviation, *NIHSS* National Institutes of Health Stroke Scale, *RSSI* recent small subcortical infarcts, *WMH* white matter hyperintensities, *IQR* interquartile range, *sGFAP* serum glial fibrillary acidic protein, *mRS* modified Rankin Scale

^a^New infarcts or progression of WMH on follow-up MRI scans

### Evolution of sGFAP levels over time

sGFAP measurements were available in 90 patients at the 3-months and in 88 patients at the 15-months follow-up. At both timepoints, sGFAP levels were lower compared to baseline (*p* < 0.001, respectively) and did not differ from control levels (Table 1, Fig. 1).

In univariable analysis, median sGFAP levels at both follow-ups were higher in patients with more severe WMH according to Fazekas scores 2 or 3 (follow-up 1: 133.4 vs. 92.2, *p* = 0.006; follow-up 2: 139.3 vs. 92.6 pg/ml, *p *< 0.001), presence of lacunes (follow-up 1: 131.8 vs. 113.7, *p* = 0.04; follow-up 2: 133.8 vs. 105.8 pg/ml, *p* = 0.02) and microbleeds (follow-up 1: 149.9 vs. 113.7, *p* = 0.09; follow-up 2: 166.6 vs. 111.2 pg/ml, *p* = 0.04) at the respective follow-up MRI scans. However, this association was no longer present after adjusting for age, sex and arterial hypertension (*p* > 0.05, respectively). sGFAP levels were also not related to WMH severity and presence of chronic CSVD features (lacunes, microbleeds) in the 53 controls (*p* > 0.05, respectively).

During the entire 15-months follow-up period, 14 patients had new vascular brain lesions on follow-up MRI (12 had progression of CSVD with 2 recurrent symptomatic RSSI, 7 new silent lacunes, 3 new WMH; 2 patients had incident cortical infarcts). Progressive cerebrovascular disease (new vascular brain lesions on follow-up MRI) was neither related to sGFAP levels at baseline (*p* = 0.10), nor at follow-up 1 (*p* = 0.86) or 2 (*p* = 0.20). Associations between sGFAP levels and new vascular brain lesions separately shown for both follow-up MRI investigations are provided in Table 3.

**Table 3:** Serum GFAP levels in relation to new cerebrovascular lesions on follow-up MRI

3 months follow-up (*n* = 90)	No new vascular MRI lesion at 3 months (*n* = 83)	New vascular MRI lesion at 3 months (*n* = 7)	*p* value
Baseline sGFAP	186.3 (246.4)	141.4 (123.5)	0.53
3-month sGFAP	116.2 (100.2)	145.5 (100.5)	0.52
15-month sGFAP	112.9 (95)	137.9 (133.2)	0.38

15-month follow-up (*n* = 88)	No new vascular MRI lesion at 15 months (*n* = 76)	New vascular MRI lesion at 15 months (*n* = 12)	
Baseline sGFAP	189.9 (260.8)	127.9 (121.8)	0.14
3-month sGFAP	115.0 (100.7)	133.4 (103.2)	0.77
15-month sGFAP	115.2 (96.6)	121.2 (103.3)	0.20

sGFAP levels indicated in median (interquartile range)

*sGFAP* serum glial fibrillary acidic protein

Moreover, neither baseline nor follow-up sGFAP levels were associated with functional neurological outcome according to the mRS (*p* > 0.05, respectively, Table 2).

## Discussion

In this prospective observational study, we could demonstrate that sGFAP quantified by the ultrasensitive SIMOA technique in the first days after stroke symptom onset is increased in patients with RSSI compared to age- and sex-matched controls with comparable WMH severity. While sGFAP further correlated with the size of the RSSI, this marker was not associated with chronic CSVD features or the occurrence of incident cerebrovascular lesions during a follow-up period of 15 months. These findings indicate that serum GFAP is a sensitive marker for acute tissue damage even in small subcortical brain infarction but not related to chronic brain damage from CSVD as evidenced by MRI.

Our work provides novel information as GFAP has not yet been investigated in sporadic CSVD and lacunar stroke (with its neuroimaging correlate RSSI) as the most destructive phenotype of CSVD. There exists only one prior report on the role of sGFAP in CADASIL patients [7]. In this work, sGFAP levels were increased in 63 Taiwanese CADASIL patients compared to 17 controls. In line with our findings, sGFAP was not associated with the severity of WMH and lacunes, but in contrast to our work, sGFAP was higher in patients with cerebral microbleeds, and predicted incident intracerebral hemorrhage over 3 years of follow-up. In this context, it is important to note that a direct comparison between genetic and sporadic CSVD is limited due to various pathophysiological differences. Moreover, the two study cohorts also differed regarding clinical presentations. All our patients had a sudden cerebrovascular event (lacunar stroke with MRI-confirmed RSSI), while the CADASIL patients represented a rather heterogenous group with prior ischemic or hemorrhagic strokes or even without a history of stroke, and they had more extensive CSVD with more lacunes and especially microbleeds on brain MRI. Given these methodological constraints, the Taiwanese study [7] also remained unresolved whether sGFAP is already increased in the acute phase of CSVD-related stroke, as blood sampling for sGFAP measurement was performed more than one year after stroke symptom onset. Furthermore, prior studies on serum sGFAP in stroke mostly used conventional ELISA techniques (as opposed to the ultrasensitive SIMOA assay applied in our work), analyzed more heterogenous cohorts of acute ischemic stroke patients with more severe strokes and mainly concentrated on the differentiation between ischemic and hemorrhagic stroke subtypes, thereby consistently showing higher GFAP levels in patients with acute intracerebral hemorrhage [2–7, 15, 16]. Moreover, none of these studies accounted for concomitant chronic cerebrovascular lesions or lesion progression on follow-up MRI scans and they also did not provide serial sGFAP measurement available for the analysis of temporal dynamics of this biomarker after the acute event. While we found a positive correlation of sGFAP with axial RSSI diameter, suggesting that sGFAP levels increase with the size of ischemic infarction, neither baseline nor follow-up GFAP levels were related to clinical stroke severity (according to the NIHSS) or functional neurological outcome (rated by the mRS). These findings contrast the results from an earlier study in Chinese stroke patients [6], reporting that sGFAP levels measured one day after stroke symptom onset were increased in patients with higher NIHSS scores and associated with worse functional neurological outcome at 1 year. However, we acknowledge that the range of NIHSS and mRS in our cohort might have been too small to detect such a potential effect in our work.

Because of the dedicated design of our study, we also were able to investigate the temporal dynamics of sGFAP levels in RSSI. Interestingly, there was no correlation of sGFAP levels with the time from symptom onset to baseline blood sampling within 13 days, even after adjusting for age, sex and RSSI size. This observation indicates that sGFAP is a sensitive acute marker for small ischemic infarcts that is rapidly released into the blood. Blood brain barrier dysfunction and alterations of the glymphatic system—two mechanisms which have been implicated in the pathophysiology of CSVD—might accelerate GFAP drainage into the blood and might contribute to its rapid detection in the serum of RSSI patients [17, 18].

While our findings potentially indicate that sGFAP stays elevated for at least about 2 weeks after RSSI onset (given the lack of change with time from acute symptoms to sampling), our work additionally demonstrated that sGFAP levels return to the levels observed in controls at 3 months after stroke and that they remain at this level 15 months after the stroke incident. This suggests that a rise in sGFAP reflects acute astro-glial damage rather than ongoing injury and/or activation. As a matter of fact, our study cannot serve to define the exact timepoint when sGFAP levels peaked or started to decrease, because this would necessitate high frequency blood sampling in narrow time intervals. In this context, a recent review [2] identified that there is a clear knowledge gap regarding longitudinal dynamics of sGFAP in (sub)acute neurological conditions. In a small study on 34 patients with traumatic brain injury, sGFAP levels were highest on day 1 but still elevated at 90 days after the acute event [19]. This difference compared to our findings can be attributed to the more severe global and diffuse cerebral damage and blood barrier disruption in cerebral trauma.

Although follow-up sGFAP levels were associated with WMH severity as well as presence of lacunes and microbleeds in univariable analysis, these relationships were no longer present after adjusting for the important covariates age, sex and arterial hypertension. The missing association of sGFAP values and chronic CSVD neuroimaging features in our control participants points into the same direction.

In a substantial number of patients, CSVD has a progressive disease course with the occurrence of new cerebrovascular lesions which often occur without obvious clinical symptoms but—as they accumulate—associate with cognitive impairment and dementia, gait disturbance, depression and an increased rate of recurrent stroke, brain hemorrhage and mortality [20–22]. Because frequent follow-up MRI scans to capture progressive CSVD are not feasible, a blood-derived biomarker for this purpose would be highly desired. Unfortunately, our results argue against such a potential role for GFAP, at least over a follow-up period of 15 months. Notably, we cannot rule out that GFAP might have the potential to indicate microscopic damage that remains undetected by conventional MRI sequences. Such an association has been described in patients with mild traumatic brain injury [23] and should be a target for future studies on CSVD (e.g., using diffusion tensor imaging or other techniques to capture ultastructural tissue damage which can precede obvious lesion detectable by routine brain MRI.

Considering the given limitations, our findings do not suggest that sGFAP may serve as a tool to identify (clinically silent) CSVD progression. On the other hand, there might have been too few events and the time period might have been too long to capture the (acute) occurrence of new cerebrovascular lesions with increased GFAP levels. We do not know the exact timepoint when the clinically silent lesions occurred in our patients, and therefore, tissue destruction together with GFAP levels might have already stabilized at time of blood sampling.

Notably, serum neurofilament light that has also been investigated in a subgroup of the present study cohort in an earlier work [8], was not only elevated in the acute phase of RSSI but also correlated with WMH severity and progressive CSVD on follow-up brain MRI. Therefore, the neuroaxonal biomarker neurofilament light seems to be more sensitive for ongoing (clinically silent) active CSVD compared to sGFAP, which—if confirmed in further independent cohorts—might have the potential to serve as an acute marker even for small subcortical ischemic brain infarcts.

Major strengths of our study include the application of the ultrasensitive SIMOA technique for sGFAP assessment and the availability of longitudinal GFAP measurements. To minimize the impact of concomitant neurodegenerative processes which could have affected sGFAP levels [2, 24], we used an age limit above 75 years and an mRS score of > 1 as exclusion criteria in this study (enabling analysis of sGFAP in more “pure” CSVD). However, we acknowledge that our cohort might not represent the entire spectrum of patients with lacunar stroke.

Another limitation of this study comes from the fact that it was not primarily designed to study the role of GFAP as a biomarker. This explains our focus on patients with an RSSI and the different sampling intervals. The sample size of the present study was moderate and consistent measurements of GFAP were missing in a few patients. In this context, we were not able to identify factors that could explain the wide range of sGFAP levels in our cohort.

On the other hand, the availability of longitudinal information on GFAP levels and the comprehensive and standardized MRI follow-up scans at predefined timepoints allowing for exact identification of neuroimaging markers of CSVD and their progress over time represent major strengths of the present work.
